# The Effect of Calendula Officinalis in Therapy of Acetic Acid Induced Ulcerative Colitis in Dog as an Animal Model

**Published:** 2011-12-01

**Authors:** D Mehrabani, M Ziaei, S V Hosseini, L Ghahramani, A M Bananzadeh, M J Ashraf, A Amini, M Amini, N Tanideh

**Affiliations:** 1 Stem Cell and Transgenic Technology Research Center, Shiraz University of Medical Sciences, Shiraz, Iran; 2 Laparascopy Research Center, Shiraz University of Medical Sciences, Shiraz, Iran; 3 Colorectal Disease Research Center, Shiraz University of Medical Sciences, Shiraz, Iran; 4 Department of Anesthesiology, Shiraz University of Medical Sciences, Shiraz, Iran

**Keywords:** Ulcerative colitis, Acetic acid, Calendula officinalis, Therapy, Dog

## Abstract

**Background:**

In patients with ulcerative colitis (UC), the repeated cycle of injury and repair of intestinal mucosa has been reported to increase the risk of colon cancer. So, a safe and efficient therapy is required for the treatment and prophylaxis for the disease.This study aims to investigate the efficacy of Calendula officinalis extract in treatment of experimentally induced ulcerative colitis in dog animal model.

**Methods:**

During fall 2010, 10 out-bred female German dogs (1-2 years old; weighs of 20-25 kg) were enrolled. Ulcerative colitis was induced with 6% acetic acid as enema and method of treatment was retrograde (via enema) too by C. officinalis.

**Results:**

Loose stools, diarrhea, gross bleeding and loss of body weight happened after administration of acetic acid and crypt damage, loss of epithelium, infiltration of inflammatory cells and depletion of goblet cells were noticed histologically. C. officinalis could successfully resolve the damages of UC.

**Conclusion:**

Treatment with C. officinalis can broaden the current therapy options for UC.

## Introduction

Inflammatory bowel diseases (IBD) are chronic fluctuating inflammatory diseases with unknown clear causes; believed to be multifactorial and challenging in management of the disease. IBD has divided into two major group including Ulcerative colitis (UC) that involves the mucosa of the rectum and colon and Crohn's disease (CD). Patients with UC complain from bloody diarrhea, abdominal pain, weight loss, chronic pain, side effect of drug management and especially in long term, colon cancer related signs. UC is idiopathic, chronic, relapsing and its inflammatory condition is immunologically mediated in the lining of the rectum and colon. In adults, UC is most commonly diagnosed between the third and fourth decades of life, with no difference between males and females.[1]

A decrease in antioxidant defense against increasing oxidative materials have been revealed in colonic mucosal biopsies of patients with UC.[2] The therapeutic strategy for UC has focused on anti-inflammatory agents.[3] Glucocoticoids and salicylates as well as biological agents against tumor necrosis factor-α (TNF-α) were used in treatments of UC.[4]Corticosteroids as another therapeutic measure are potent inhibitors of T-cell activation and pro-inflammatory cytokines,[5] while fewer side effects were shown for steroids used via enema compared to a systemic use.[6]

Calendula officinalis L. known as calendula is a flower from the Asteraceae family has several medical properties including bactericidal, antiseptic, antiinflammatory and analgesic entities.[7] The petals and pollen contain triterpenoid esters (an anti-inflammatory property) and the carotenoids flavoxanthin and auroxanthin (an antioxidant property and the source of its yellow- orange coloration).[8] The leaves and stems contain other carotenoids, mostly lutein (80%) and zeaxanthin (5%), and beta-carotene and its extract contains saponins, resins and essential oils[7][9] that enhance the level of endogenous anti-oxidant catalases, elismutases and glutathione having anti-oxidant effects.[10] C. officinalis has been successfully used for preventing UV-induced oxidative stresses,[11] melanoma metastasis control,[12] improvement of wound healing processes[13] and angiogenesis activation.[14]

Several studies have used herbal extracts for treatment of IBD.[14][15] In this regards, Medhi et al. demonstrated that Manuka honey has synergic effects with sulfasalazine in enhancing antioxidant defense system in experimentally induced UC model in rats.[14] Souza et al. showed that enemas of budesonide and probiotics enhance the mucosal trophism in experimental colitis in rats.[16] According to anti-inflammatory entities of C. officinalis and lack of data regarding its efficacy in treatment of UC, this study was performed to investigate the efficacy of C. officinalis extract in treatment of experimentally induced UC in a dog model.

## Materials and Methods

In this experimental study being performed during fall 2010 in Laboratory Animal Center of Shiraz University of Medical Sciences, 10 out-bred female German dogs (1-2 years old; weighs of 20-25 kg) were enrolled. All dogs were housed individually and fed standard food throughout the experiment. The dogs were initially evaluated for any illness by physical examination and laboratory screening. All experiments were carried out under aseptic conditions and the protocol of anesthesia; surgical procedures, postoperative care and sacrifice were identical for all animals The study was approved by the Ethics Committee of Shiraz University of Medical Sciences (registration number: 90-5077). Study animals were handled in conformity with guidelines for the care and handling of laboratory animals provided by Shiraz Laboratory Animals Center. The dogs were randomly allocated to two study groups (5 in each group) to receive C. officinalis extract or saline enema as treatment. A 40% C. officinalis solusion was prepared by Pharmacology Department of Shiraz University of Medical Sciences.

Ulcerative colitis was induced in all the dogs. For this purpose animals were kept NPO for two days and bowel preparation was performed for all of them on the day of induction. Bowel preparation was done using 30 ml of Senagraph syrup (Sina Daru, Tehran, Iran) through a nasogastric tube. To check for presence of ulcers in the colon, 0.1 mg/kg of acepromazine was administered. Only water was given ad libitum to animals during these two days. Ulcerative colitis was induced by acetic acid 6% (6 mg/kg/stat) enema through rectal tube.

After 7 days of enema, the colon was evaluated by a rigid rectosigmoidoscope to evaluate any presence of any gross mucosal ulcer as described by Morris et al. (1994)[17] (Table 1). Multiple biopsies were provided from mucosa in 10, 20 and 30 cm proximal to the anal verge and transferred into the formalin for histological studies (H and E staining) as described by Mehrabani et al. (2009)[18] (Table 2). In histological study, the samples were examined at x10 and x20 magnification for presence or absence of ulcer, mucosal cell depletion, inflammatory cysts, congestion, mucosal atrophy, submucosal edema, inflammatory cells, vascular dilatation, etc. At magnification of x40, a histological scoring was made from 20 random fields per section from each specimen (Table 3). After histological confirmation of induction of UC in all animals, they were randomly divided into two groups. Group A received C. officinalis extract via enema (40% solution, 3 mL/day until 30 days) and Group B that received a saline enema (3 mL/day).

An identical rigid rectosigmoidoscopy was repeated after 14 and 30 days of UC induction to provide multiple biopsies from the mucosa for histological study. After 45 days, another rigid rectosigmoidoscopy was performed to take biopsies for histological evaluation. According to the criteria described by Dundar et al. (2008) for severity of colitis, all dogs were scored.[19] For histological study, the animals were sacrificed with an overdose of anesthetics. All statistical analyses were performed with the Statistical Package for Social Sciences version 16.0 (SPSS Inc., Chicago, Ill., USA). Chi-square test was used to compare the proportions between groups. A p value of less than 0.05 was considered statistically significant.

**Table 1:** Criteria for scoring of macroscopic damage^a^.

**Score**	**Macroscopic morphology**
0	No damage
1	Localized hyperemia, but no ulcer
2	Linear ulcers with no significant inflammation
3	Linear ulcer with inflammation at one site
4	Two or more sites of ulceration and/or inflammation
5	Two or more major sites of inflammation and ulceration or one major site inflammation and ulceration extending 41 cm along the length of the colon

^a^ An Inflammation was defined as regions of hyperemia and bowel wall thickening.

**Table 2:** The variables used for microscopic scoring.

**Variable**	**Severity of changes**			
	**0**	**1**	**2**	**3**
Ulceration	No ulcer	Erosion or single ulceration not exceeding lamina muscularis mucosa	Multifocal ulcerations not exceeding the submucosa	Ulcerations exceeding the submucosa
Mucus cell depletion	Preserved mucus cell	Mild depletion in a few cells	Moderate depletion (<50% of cells)	Severe depletion or complete disappearance of mucosa
Crypt abscesses	No abcesses	1–3 abscesses/ slide	4–9 abscesses/slide	10 or more abscesses/ slide
Inflammatory cysts	No cysts	1–3 cysts/slide	4–9 cysts/slide	10 or more cysts/slide
Mucosal atrophy	Normal thickness	Mild atrophy (<10%)	Moderate atrophy (10–50%)	Severe atrophy (<50%)
Edema (submucosa)	Normal thickness	Mild edema (submucosal Expansiono< 10%)	Moderate edema (submucosal expansion, 10–100%)	Severe edema (submucosal Expansion> 100%)
Inflammatory cell infiltration	No inflammatory cell infiltration	Mild inflammatory cell infiltration	Moderate (distributed but not dense) inflammatory cell	Dense inflammatory cell infiltration
Vascular dilatation	Normal blood vessels	Mild dilatation of single blood vessel	Moderate dilatation of several blood vessels	Severe dilatation of several blood vessels

**Table 3:** Results of treatment in group A.

**Variable**	**After 7 days (Before treatment)**					**After 14 days**					**After 30 days (p=0.04)**					**After 45 days (p=0.005)**				
	**1**	**2**	**3**	**4**	**5**	**1**	**2**	**3**	**4**	**5**	**1**	**2**	**3**	**4**	**5**	**1**	**2**	**3**	**4**	**5**
Ulceration	0	1	1	0	0	0	0	1	0	0	0	0	0	0	0	0	0	0	0	0
Mucous cell depletion	0	1	1	2	2	0	1	0	1	1	0	1	0	0	0	0	0	0	0	0
Crypt abscess	1	1	1	1	1	0	1	1	1	1	0	0	0	0	1	0	0	0	0	0
Inflammatory cyst	0	0	0	0	0	0	0	0	0	0	0	0	0	0	0	0	0	0	0	0
Mucosal atrophy	1	1	1	1	1	0	0	1	1	1	0	0	0	0	0	0	0	0	0	0
Submucosal edema	1	1	1	1	1	0	0	1	1	1	0	0	0	0	1	0	0	0	0	0
Inflammatory cell	2	2	3	2	2	1	1	2	2	2	1	1	1	1	1	0	0	0	0	0
Vascular dilatation	2	2	2	2	2	1	2	2	2	2	1	2	2	2	1	0	0	0	0	0
Total	7	9	10	9	9	3	5	7	8	8	2	4	3	3	4	0	0	0	0	0

## Results

**Fig. 1:**
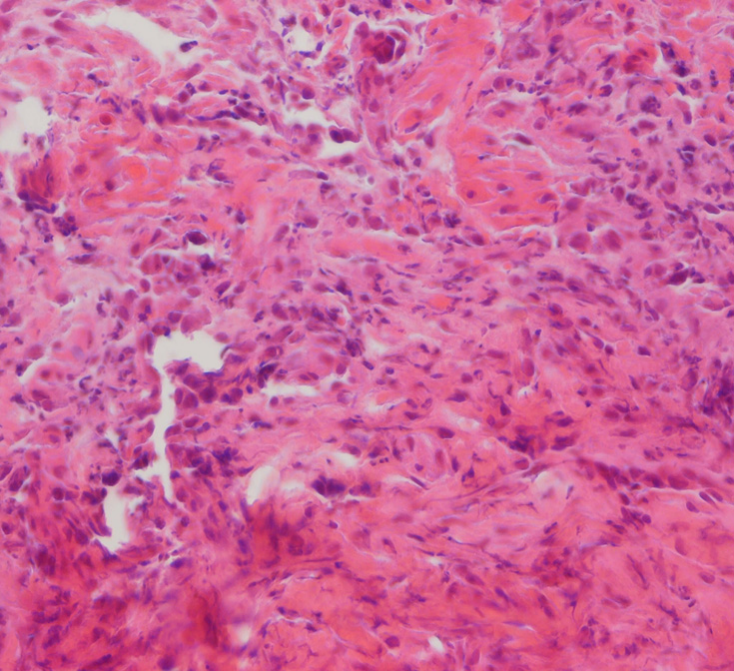
Severe inflammation, PMN infiltration in lamina propria, glandular destruction and goblet cell depletion denoting to UC after 7 days (x400, H and E).

**Fig. 2:**
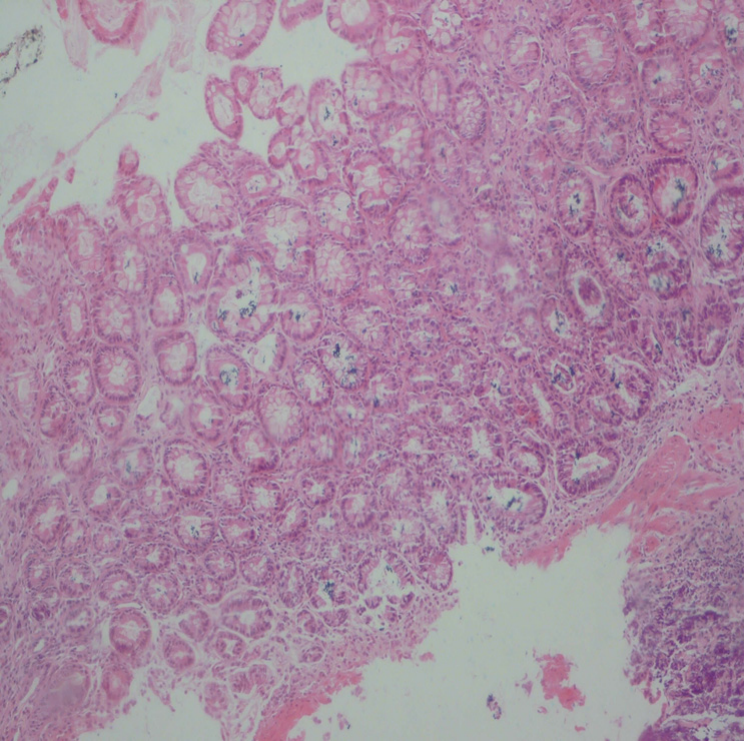
Mild PMN infiltration and goblet cell depletion after 30 days (x100, H and E).

**Fig. 3:**
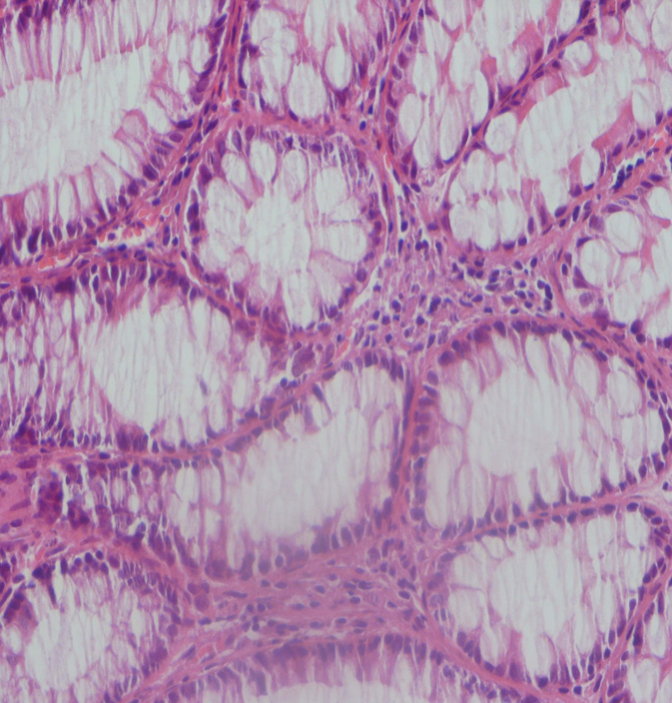
Complete healing after 45 days of treatment (x400, H and E).

In dogs received 6 ml/kg of 6% acetic acid for two days, typical changes related to ulcerative colitis e.g. multiple ulcers and diffuse inflammation were noticed with a score of 5. After 7 days, the biopsies revealed inflammatory cells in mucosa and around the crypts that were polymorphonuclear leukocytes and lymphocytes. Multiple ulcerations were also noticed indicating to presence of a crypt abscess. In the submucosa, multifocal areas of inflammation and ulceration were present and it was diffusely edematous. Infiltration of polymorphonuclear leukocytes, eosinophils and lymphocytes was extensive. Figure 1 demonstrates a severe inflammation, PMN infiltration in lamina propria, glandular destruction and goblet cell depletion denoting to UC after 7 days (x400, H and E); Figure 2 shows a mild PMN infiltration and goblet cell depletion after 30 days (x100, H and E) and Figure 3 denotes to a complete healing after 45 days (x400, H and E).

Table 3 shows the findings of retrograde treatment by C. officinalis in dogs. On day 7th, the scoring was 7-10, on day 14th was 3-8, on day 30th was 2-4 and 0 on 45th day.

On days 14th (p>0.05), 30th (p=0.04) and 45th (p=0.005), the mucosal healing was statistically significant compared to the 7th and 14th day.

## Discussion

Many reports showed that hamsters, mice or rats as experimental models of UC.[20][21][22][23] Administration of 6% acetic acid into the colon led to a fast development of severe inflammation and ulceration of the colon together with diarrhea, hematochezia and weight loss that are identical to what occurs in human UC. In this study, macroscopic findings were ulceration and hemorrhage. Microscopic findings were an increase in infiltration of polymorphonuclear leukocytes, lymphocyte and presence of cryptic abscesses similar to human UC.

Regarding UC induction, there are several reports on different chemicals to induce experimental colitis such as acetic acid, 2,4,6-trinitrobenzene sulfonic acid, dextran sodium sulfate, oxazolone and indomethacin.[24] In this study, 6% acetic acid was used for induction of UC. It is easily available and an inexpensive chemical agent. Our findings confirmed induction of UC sigmoidoscopically and histologically.

Many methods of treatment of UC are used in experimental animal models such as corticosteroids, salazosulfapyridine, azathioprine, mesalazine, methotrexate, 6-mercaptopurine, and cyclosporin.[25]

They are mostly targeted to reduce symptoms of UC. However clinically, the therapeutic measure that can prevent a relapse may also be as important as a treatment to decrease symptoms.[26] They demonstrated that fibratide decreased the severity of experimental dextran sulfate sodium (DSS)-induced UC in mice and reported that it can be potentially beneficial in treatment of inflammatory bowel diseases (IBD). Topical use of GM-CSF on mucositis reduced the duration of healing time via suppressing inflammatory reaction and proliferating epithelia.[20] Tozaki et al. showed that chitosan capsules were useful carriers in colon-specific delivery of anti-inflammatory drugs such as 5-ASA and the healing of TNBSinduced colitis in rats.[27] Treatment with ECP antibody was shown to improve DSS-induced colitis in rats, probably because of increased regenerative activity of the colonic epithelium and downregulation of the immune response. Therefore, anti-ECP may promote intestinal wound healing in patients with UC.[23]

The colonic mucosal layer was shown as a potential therapeutic target for IBD based on the histological findings that in IBD patients, a thinner mucosal layer and depletion of goblet cells in the colonic epithelium are seen.[28] The mucosal layer is mostly composed of mucins and acts as a physical barrier to protect the epithelium from agents disturbing epithelium integrity and may also prevent the intestinal microflora from triggering abnormal immune responses.[28] They Showed that in mice with disrupted mucus synthesis suffered from more serious colitis, presence of mucus is important in inflammation.[28] An injury in the gastrointestinal tissue is usually associated with healing needing the production of granulation tissue including deposition of connective tissue matrix, proliferations of fibroblasts and angiogenesis for reconstruction of mucosal microvessels that are critical for delivery of oxygen and nutrients to the healing site.[28] In last step of healing, re-epithelialization and reconstruction of epithelial structures occurs.[29]

After development of injury in the mucosal layer, the intestinal epithelium would reestablish fast its integrity. To reestablish the integrity of the mucosal layer, epithelial cells migrate into the wounded area (epithelial restitution) and would later proliferate to replace the decrease in the cell pool. A variety of soluble peptides, growth factors, prostaglandins and cytokines are also secreted in a coordinated fashion in the injured area to restore mucosal integrity.[30]

Calendula officinalis is a herb having medicinal properties containing biologically active complex substances of Carotin (Provitamin A), Stearin, Triterpiniod, Plavonoid, Kumarin, macro and micro compound elements.[31] There are several reports on different properties of Calendula officinalis L. showing that this herbal is effective in protection against subacute ciggaret smoking-induced cell injury,[32] has antimetastatic effects,[33] antiparasitic and antibacterial efficacies, [34][35] spasmolytic properties,[36] wound healing and anti-inflammatory properties,[37][38] hepato- and renoprotective action,[39] and Angiogenic activity.[14]There are no data available on anti ulcer effect of this herbal in IBDs. Our findings denote to a significant mucosal healing after administration of C. officinalis after 30 and 45 days.

We showed that our therapy could well resolve the damages. So C. officinalis may provide an opportunity for UC therapy and broaden the current treatment choices.
